# The Relationship Between Stiff Knee Gait Runner’s Dystonia and Musculoskeletal Knee Pathology: A Case Series

**DOI:** 10.3390/toxins17030121

**Published:** 2025-03-03

**Authors:** Jared A. Stowers, Derek S. Day, Steven Jow, Sarah Heins, Euan Forrest, Yonathan M. Assefa, Paige M. Lind, Afreen Mushtaheed, Frances T. Sheehan, Katharine E. Alter

**Affiliations:** 1 MedStar Georgetown National Rehabilitation Hospital, Washington, DC 20010, USA; 2 Department of Physical Medicine and Rehabilitation, University of Utah Health, Salt Lake City, UT 84132, USA; 3 Holmdel Sports and Spine, Holmdel, NJ 07733, USA; 4 Department of Undergraduate Medical Education, Georgetown University School of Medicine, Washington, DC 20007, USA; sjh127@georgetown.edu; 5 The Department of Rehabilitation Medicine, National Institutes of Health, Bethesda, MD 20892, USAafreen.mushtaheed@nih.gov (A.M.); gavellif@cc.nih.gov (F.T.S.); kalter@cc.nih.gov (K.E.A.)

**Keywords:** knee pathology, botulinum neurotoxin, BoNT, ultrasound, US

## Abstract

Background: Runner’s dystonia (RD), a rare task-specific lower-limb dystonia affecting high-mileage runners, presents as abnormal lower-extremity muscle contractions during running. Treatment of RD is challenging and often confounded by significant diagnostic delays due to overlapping symptomatology with other conditions. This case series examines the relationship between stiff knee gait RD and musculoskeletal (MSK) knee pathology. Methods: Eight RD cases, evaluated at the NIH Movement Disorders Clinic since 2018, were retrospectively reviewed. Patients underwent neurological, biomechanical, and MSK evaluations, including 3D motion analysis, surface electromyography, and knee ultrasound. Therapeutic interventions, including botulinum neurotoxin (BoNT) injections, were assessed. Results: Seven patients demonstrated stiff knee gait subtypes, with all having ipsilateral and/or contralateral knee effusions or tendinopathies. Three patients who received MSK interventions (e.g., aspiration, corticosteroid injections) combined with BoNT therapy experienced significant symptom improvement. One patient with isolated foot dystonia displayed different biomechanical patterns without knee pathology. Conclusions: RD patients with stiff knee gait often exhibit knee pathology, most likely due to altered biomechanics and running history. Addressing both issues is essential for optimizing treatment outcomes, reducing pain, and improving function, especially since pain can trigger dystonia. Future research should determine the ideal sequence of interventions for RD patients with MSK issues to develop effective, personalized treatment algorithms.

## 1. Introduction

Runner’s dystonia (RD), a rare task-specific lower-limb dystonia affecting high-mileage runners, presents as abnormal lower-extremity muscle contractions during running [1,2,3]. It is a unique, typically idiopathic phenotypic subtype of task-specific lower-limb dystonia and is less extensively described in the literature compared to other dystonic conditions [4,5]. Focal lower-limb dystonias are the least common form of focal dystonia and may represent the first symptom of other neuromuscular disorders (e.g., Parkinson’s disease) [6,7]. As such, patients with RD are likely to be under or misdiagnosed, leading to unnecessary procedures, risks, expenses, and delays in initiating effective treatment [3].

RD tends to affect the lower limbs or trunk, and typically presents in adulthood, most often in long-distance, high-mileage elite runners. The initial symptoms manifest during running and may later generalize to affect forward, but not backward, walking [8]. This differentiates RD from other task-specific dystonias, which rarely generalize to affect other tasks [3]. RD can involve multiple muscle groups and span adjacent anatomical regions or joints. However, the muscles involved in the RD dystonic pattern rarely generalize to different muscle groups beyond their focal or segmental area [4].

Historically, RD has been considered a diagnosis of exclusion, requiring a thorough neurological and musculoskeletal (MSK) history and examination with a detailed functional assessment. Initial diagnostic tests often include plain radiographs, brain/spine magnetic resonance imaging (MRI), and other neuroimaging and electrodiagnostic (EDX) tests before a formal diagnosis can be established. When utilized appropriately, surface electromyography (sEMG), in combination with a time-lock kinematic assessment using 3D computerized motion capture technology, is particularly effective in determining dystonic muscle activation and compensatory or alternative causes of abnormal gait [4]. The non-dystonic (asymptomatic) limb/joint should be, but is not always, included in the RD evaluations, as dystonia in one limb affects joint kinematics and muscle activation in the uninvolved extremity, potentially leading to maladaptive compensatory strategies [9].

RD patterns can be characterized into biomechanically unique phenotypic subtypes based on the joint(s) involved (Table 1). Each RD subtype requires a detailed understanding of the underlying neurophysiology for appropriate treatment and management. For example, the “stiff knee” gait subtype is caused by a diminished ability to initiate or control knee flexion or extension (KF or KE) during the stance or swing phase of the gait cycle [10]. This RD subtype has several potential causes, including hamstring dystonia, which causes a stiff KF gait, and quadriceps dystonia, leading to a stiff KE gait. Another possible cause of stiff knee gait pattern is abnormal timing/duration of plantarflexion (**PF**) activation (dystonia) during the stance or swing phases. This leads to insufficient foot/ankle dorsiflexion during stance or at toe-off. Stance-phase PF mistiming leads to aberrant ground reaction forces, creating PF-KE coupling, which causes stance-phase hyperextension and limits swing-phase KF [11,12]. The inability to achieve stance-phase dorsiflexion or swing-phase KF leads to poor toe clearance and tripping [10]. Dystonia in the affected muscles leads to decreased bi-directional knee joint range of motion, altered multi-joint biomechanics, and the evolution of compensatory measures in both lower limbs.

We postulate these compensatory gait changes can lead to overuse injuries, tendinopathies, and degenerative joint conditions in the lower extremities. Thus, we hypothesize runners with stiff knee gait dystonia, which results in altered gait biomechanics, are predisposed to knee injuries or overuse syndromes due to altered knee joint forces. This manuscript details seven cases of stiff knee gait RD and one without who were evaluated in the NIH movement disorders clinic since 2018.

## 2. Case Summaries

Upon enrolling in our clinic, a complete, systematic history and physical (Table 2) was performed for each patient. This included a neurological evaluation and a 3D computerized motion analysis.

### 2.1. PF-KE Couple-Predominant (Cases 1–4):

**Figure 1:**
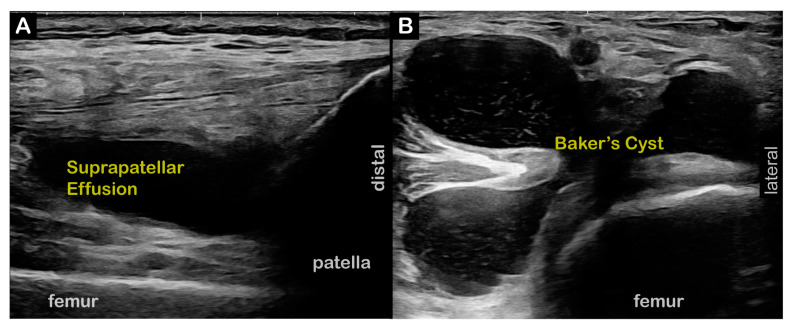
**Case 1** (**Dystonia in Right Limb)**: Suprapatellar effusion and Baker’s cyst affecting dystonic limb. (**A**) Two-dimensional long-axis image of right distal quadriceps tendon at patellar insertion. ***Pathology*** = Large suprapatellar effusion (SPE), **right**. (**B**) Two-dimensional short-axis image of right posterior knee. ***Pathology*** = Baker’s cyst, **right**.

**Case 1:** A 68-year-old male former professional long-distance runner presented at our clinic with six years of right knee stiffness and progressive difficulty running. The PF-KE couple stiff knee RD subtype was confirmed in our clinic. **Time from symptoms to diagnosis**: 4 years. Two-dimensional ultrasound imaging revealed a right knee Baker’s cyst and a large lateral suprapatellar recess fluid collection (Figure 1). **Interventions**: The patient failed an initial trial of levodopa/carbidopa. Prior to initiating BoNT injections, the patient was referred for aspiration of the fluid collections and corticosteroid injection for a popliteus tear diagnosed at the time of the aspiration procedure. **Current treatment**: BoNT injections into the right medial and lateral gastrocnemius and soleus muscles and articulated AFO with plantarflexion stop. **Outcomes**: The patient demonstrated mild-to-moderate improvements in walking but has not returned to running. He cycles without symptoms.

**Case 2:** A 63-year-old female recreational runner/walker presented to NIH after 10+ years of progressive difficulty clearing her right foot and toes while walking. The PF-KE couple stiff knee RD subtype was confirmed in our clinic. **Time from symptoms to diagnosis**: 8 years. Two-dimensional ultrasound imaging revealed a left knee suprapatellar recess effusion and a Baker’s cyst. **Interventions**: The patient failed an initial trial of levodopa/carbidopa. Prior to initiating BoNT injections, the patient underwent US-guided aspiration of her Baker’s cyst, as well as diagnostic lidocaine motor point blocks to her left vastus lateralis and rectus femoris muscles. **Current Treatment**: BoNT injections to the left plantar flexor and tibialis posterior muscles. **Outcomes**: BoNT injections have led to mild-to-moderate improvement in walking, but not running, with increased speed, but limited distance.

**Figure 2:**
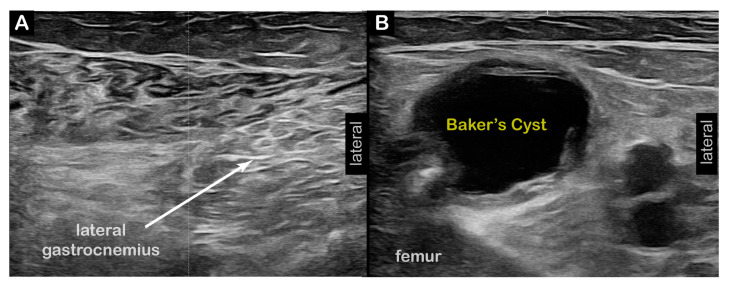
**Case 3** (**Dystonia in Left Limb):** Absent pathology in dystonic limb. Baker’s cyst is affecting contralateral extremity. (**A**) Two-dimensional short-axis image of left posterior knee; no pathology. (**B**) Two-dimensional short-axis image of right posterior knee. ***Pathology*** = Baker’s cyst, **right**.

**Case 3:** A 59-year-old female moderate-distance runner presented to NIH after 5 years of progressive left leg dragging, gait instability, and tripping while running. The PF-KE couple stiff knee RD subtype was confirmed in our clinic. **Time from symptoms to diagnosis**: 1.5 years. Two-dimensional ultrasound imaging was remarkable for a large Baker’s cyst in the right (contralateral) knee (Figure 2). There were no significant findings in the dystonic left lower limb. **Interventions**: Repair of a torn left medial meniscus. Diagnostic lidocaine motor point blocks were performed to the left gastrocnemius, soleus, flexor digitorum longus, and tibialis posterior muscles with improved walking gait. **Current Treatment**: BoNT injections to the left gastrocnemius, soleus, flexor digitorum longus, and tibialis posterior muscles. **Outcomes**: The patient reports excellent benefits, with improvements in both walking and running gaits with ongoing BoNT injections every 4–5 months.

**Case 4**: A 57-year-old female marathon runner presented to NIH after 2 years of progressive difficulty clearing her left foot while running. The PF-KE couple stiff knee RD subtype was confirmed in our clinic. **Time from symptoms to diagnosis**: 2 years. Two-dimensional ultrasound imaging revealed a mild left suprapatellar recess effusion and a small Baker’s cyst. **Interventions**: A failed trial of a left foot drop AFO. **Current Treatment:** Pending initial BoNT treatment.

### 2.2. KF-Predominant (Cases 5–6):

**Figure 3:**
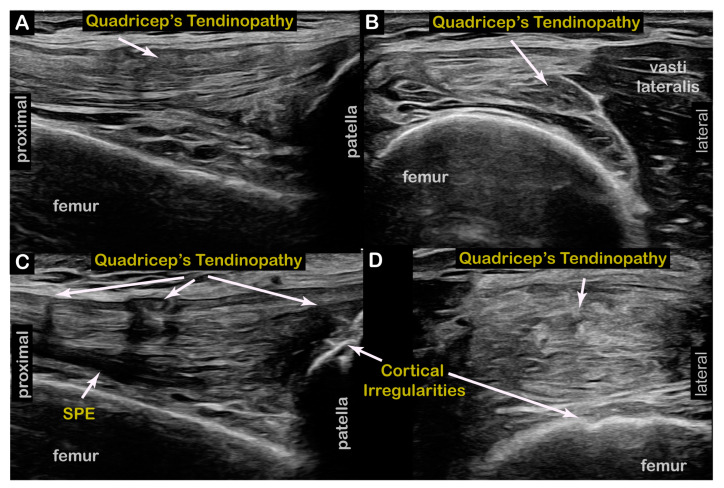
**Case 5 (Dystonia in Left Limb, Right Anterior Knee Pain)**: Bilateral distal quadriceps tendinopathy (Right > Left). (**A**) Two-dimensional long-axis image of left distal quadriceps tendon at patellar insertion. (**B**) Two-dimensional short-axis image of distal left knee. ***Pathology*** = Mild quadriceps tendinopathy, **left** (**C**) Two-dimensional long-axis image of right distal quadriceps tendon at patellar insertion. (**D**) Two-dimensional short-axis image of distal right knee. ***Pathology*** = moderately severe distal and insertional quadriceps tendinopathy and suprapatellar effusion (SPE), **right**.

**Figure 4:**
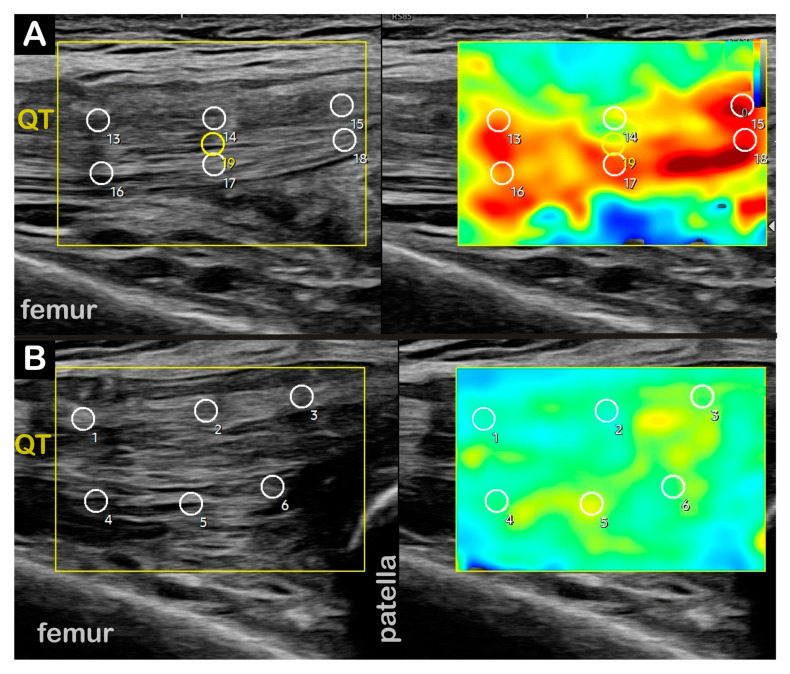
**Case 5 (Dystonia in Left Limb, Right Anterior Knee Pain)**: Shear Wave Elastography in long axis of quadriceps tendon (**A**) Left with higher stiffness (red, mild quadriceps tendinopathy; mean velocity = 7.54 m/s). (**B**) Right with decreased stiffness (yellow/green, moderately severe quadriceps tendinopathy; mean velocity = 4.27 m/s).

**Case 5:** A 74-year-old male long-distance runner presented to NIH after 5–7 years of progressive difficulty with running and limited left KE during running, leading to a shortened left leg stride length. He also reported an onset of similar, but mild, symptoms after walking short distances. The KF stiff knee-predominant RD subtype was confirmed in our clinic. **Time from symptoms to diagnosis**: 3–4 years and generalized to walking within 1–2 years. Two-dimensional ultrasound imaging revealed tendinosis of the bilateral (R > L) distal quadriceps tendons at their insertions on the superior pole of the patella (Figure 3). Shear Wave Elastography (SWE) revealed decreased stiffness of the right distal quadriceps tendon (Figure 4). **Interventions**: Failed conservative management of his gait deviations with PT, and a trial of Levodopa/Carbidopa. **Current Treatment**: BoNT injections to the left semitendinosus, semimembranosus, tibialis anterior, and adductors longus/brevis muscles. **Outcomes**: Moderate benefits with ongoing BoNT injections, with improvements in both ambulation and running gait. He is able to cycle without symptoms.

**Figure 5:**
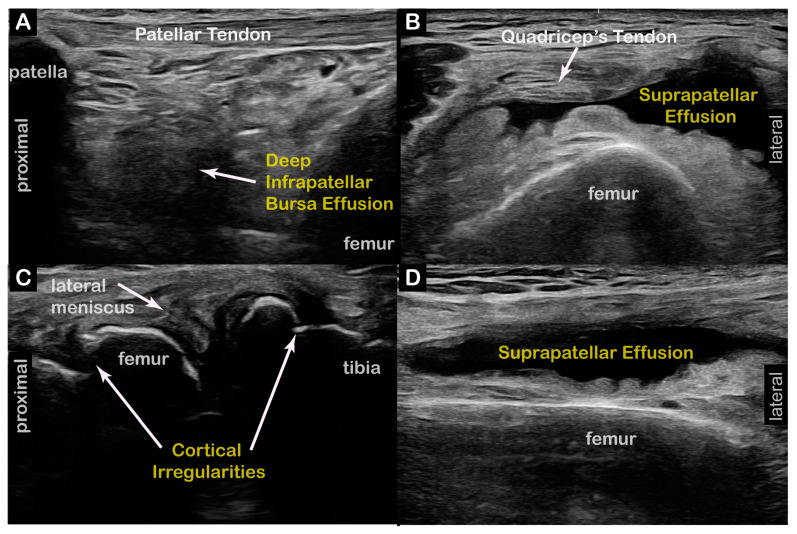
**Case 6 (Dystonia in Right Limb):** Right suprapatellar recess effusion with diffuse cortical irregularities consistent with osteoarthritis. (**A**) Two-dimensional long-axis image of right patellar tendon. ***Pathology*** = deep infrapatellar bursa effusion, **right**. (**B**) Two-dimensional short-axis image of distal quadriceps tendon. ***Pathology*** = large suprapatellar recess effusion, **right**. (**C**) Two-dimensional cross-section view of right lateral meniscus. ***Pathology*** = cortical irregularities, **right**. (**D**) Two-dimensional long-axis of distal quadriceps tendon. ***Pathology*** = large suprapatellar recess effusion, **right**.

**Case 6:** A 71-year-old male long-distance runner presented to NIH after 2 years of right lower-limb ataxia and a “peculiar gait” while walking and running. The KF-predominant RD subtype was confirmed in our clinic. **Time from symptoms to diagnosis**: 1.5 years. Two-dimensional ultrasound imaging showed a right knee suprapatellar lateral recess effusion, patellar cortical irregularity, and a deep infrapatellar effusion consistent with OA and diffuse cortical irregularities consistent with OA on the right knee (Figure 5). **Interventions**: Failed initial trials of Levodopa/Carbidopa and Trihexyphenidyl. Intra-articular right knee joint platelet-rich plasma (PRP), corticosteroid, and Hyaluronic Acid (HA) injections, with significant improvement in knee pain. **Current Treatment**: BoNT injections to the right semitendinosus, semimembranosus, biceps femoris long/short heads, and tibialis anterior, with moderate symptom improvement. **Outcomes**: Although the patient is unable to run, his walking gait has improved with BoNT injections, and he is able to swim and cycle without symptoms.

### 2.3. KE-Predominant (Case 7):

**Case 7**: A 54-year-old female long-distance runner presented to NIH after 5 years of increasing difficulty clearing her right foot/toes while running and walking. KE-predominant RD was confirmed in our clinic. **Time from symptoms to diagnosis**: 4–5 years. Two-dimensional ultrasound imaging initially showed a right suprapatellar lateral recess effusion and bilateral patellar cortical irregularities. A repeat diagnostic ultrasound was notable for a large right knee suprapatellar recess effusion and a small left knee suprapatellar recess effusion. **Interventions**: Failed PT. **Current Treatment**: BoNT injections to the right rectus femoris, vastus lateralis, and vastus medialis. **Outcomes**: The patient has experienced moderate improvements in walking gait from BoNT injections, including improved toe clearance and KF in swing. She continues to have persistent knee hyperextension in stance, and she is able to cycle without symptoms.

### 2.4. Forefoot Inversion Pattern Without PF or Stiff Knee (Case 8):

**Figure 6:**
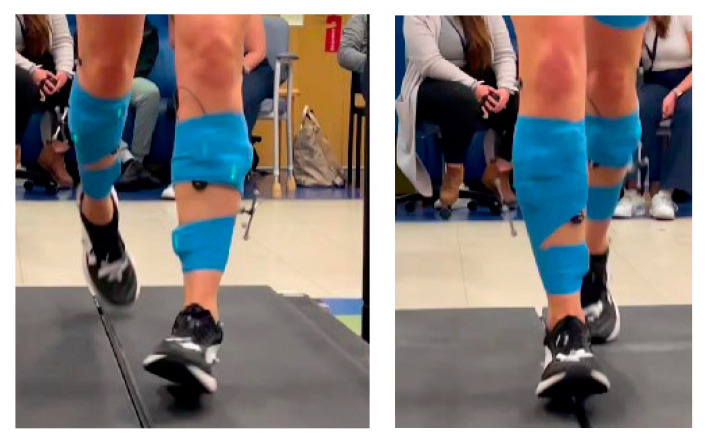
Left foot supination at initial contact compared to right foot neutral forefoot position at initial contact.

**Case 8**: A 44-year-old female runner presented to NIH after 6 years of progressive, intermittent left foot supination/inversion and toe curling when running. Excessive left food supination and inversion with toe curling were confirmed in our clinic (Figure 6). **Time from symptoms to diagnosis:** 6 years. Two-dimensional ultrasound imaging revealed no significant pathology. **Interventions:** Failed several courses of PT and gait retraining. **Current Treatment:** Excellent symptomatic improvement in running gait with BoNT injections into the left tibialis posterior and flexor digitorum longus muscles.

## 3. Results

This case series highlighted eight patients (three males and five females) diagnosed with RD. Seven were identified to have pathological stiff knee gait. Diagnostic MSK US imaging revealed pathology in the ipsilateral, contralateral, or bilateral knees of all seven patients with stiff knee gait. However, US imaging revealed differences between the sub-groups. All four patients with the PF-KE couple pattern had relatively large suprapatellar knee joint effusions, and three out of four also had large Baker’s cysts. Of the two patients with the KF pattern, one had bilateral suprapatellar effusions, but no Baker’s cyst, while the other had prominent contralateral limb quadriceps and patellar tendinopathy and mild tendinopathy in the affected limb. The lone patient with a KE-predominant pattern had a small ipsilateral suprapatellar effusion and a more considerable contralateral suprapatellar effusion.

All patients with stiff knee gait-pattern RD and knee effusion were offered a referral for a knee joint aspiration by an MSK specialist. Those with less significant effusions (Cases 2, 3, 7) elected to observe the effusion and continued BoNT injections, due to the satisfactory interventional results. Three patients with significant knee pain and pathology (Cases 1, 3, 6) underwent MSK intervention during their RD treatment course, including arthrocentesis, corticosteroid injections, PRP injections, and surgical intervention. All three experienced improved gait after their MSK intervention and continued BoNT injections (Table A1) for dystonia.

The eighth case in our series was included to illustrate the differences in gait kinematic and US findings between patients with RD affecting the plantarflexors (including foot invertors), and those with isolated foot invertor dystonia without plantarflexor involvement. While this patient presented with bilateral knee hyperextension in the stance phase, they did not present with a stiff knee during the swing phase, Furthermore, they did not demonstrate PF-KE coupling on gait analysis, or knee joint pathology on US imaging.

## 4. Discussion

This case series highlights the importance of recognizing the co-existence of dystonia and knee joint pathology in patients with RD, who appear to be predisposed to knee effusions and other concomitant knee joint pathologies which may contribute to observed altered gait patterns and influence treatment outcomes, including for BoNT therapy. Specifically, MSK impairment can lead to sub-optimal therapeutic outcomes, as these issues may impede proper joint function and, if painful, act as a trigger for the dystonic symptoms [13,14,15]. We hypothesize that acquired MSK pathology in patients with stiff knee gait RD may be attributed to a combination of dystonia-related alterations in knee biomechanics, perhaps in combination with a decades-long history of high-mileage running [16,17]. In a recent literature review, Mellinger described that elite runners are prone to developing chronic knee injuries, such as patellofemoral pain syndrome or patellar tendinopathy [18]. Runners with RD are at an even higher predisposition for developing chronic and overuse injuries, due to the repetitive maladaptive gait mechanics secondary to their dystonic muscle contractions. However, a review of previous RD case series [1,3,4,5,19,20,21,22] revealed only one previous study [19] that mentioned secondary MSK issues (e.g., two patients with Baker’s cysts were reported).

Appropriate identification and management of co-existent MSK pathology is paramount to the comprehensive management of patients with RD. This must begin with a detailed physical and functional examination of both lower extremities, to determine if the existing MSK problems may contribute to the patient’s gait deviations. Depending on the patient’s symptoms, evaluations can include imaging studies (radiographs, US, MRI, and CT) [23,24] and referral to a sports medicine physician or orthopedic surgeon. Treatment focusing only on the dystonia may lead to sub-optimal outcomes in the presence of co-existing MSK problems. While focal dystonia is typically painless, except for cervical dystonia, pain is a known trigger for increased dystonic symptoms. Therefore, unmanaged MSK-related pain may compromise treatment outcomes in RD [13]. 

Effective treatment of RD is an individualized and dynamic process, requiring a meticulous understanding of focal dystonia, functional MSK anatomy and applied biomechanics [13]. The goal of RD management is to reduce the impact of dystonia on the patient’s gait biomechanics to improve function, including participation in activities of daily living and recreational endeavors. Another treatment goal of equal importance is reducing injury risk and the acquisition of MSK problems caused by dystonia-related biomechanical gait alterations. The available RD treatments are palliative rather than curative, and standardized guidelines for MSK evaluation in patients with RD are non-existent [3,5]. While RD is relatively rare, it is likely underdiagnosed. Treatment delays of up to several years are common because RD symptoms are often misinterpreted, leading to erroneous diagnoses, such as functional neurological disorder and isolated orthopedic injury [8]. Therefore, it is essential for all clinicians who treat runners and patients with gait disorders to be familiar with RD symptoms, their presentation, and possible secondary MSK problems. This will allow for prompt diagnosis and referral for effective treatments. Early recognition of RD may reduce referrals for unnecessary, costly, and potentially invasive interventions that fail to target the primary condition.

BoNT injections are a safe and effective treatment for dystonia and are typically considered the gold-standard treatment for patients with all forms of focal dystonias, including RD (Table A1). BoNT for the treatment of RD requires that clinicians identify all muscles contributing to the patient’s gait deviations. Using US-guidance to identify key anatomical landmarks and neural distributions of highly involved muscles with precisely calculated dosages and carefully planned injections are reported to minimize adverse effects and optimize the outcomes for BoNT intramuscular chemodenervation [25,26,27,28]. A trial of oral medications (trihexyphenidyl, levodopa-carbidopa, dopamine agonists, and others) may be considered in some patients with RD, particularly those who fail to respond to BoNT injections. Still, for most patients, the benefits of oral medications are limited. The evidence for more invasive neurosurgical interventions, such as deep brain surgery (**DBS**), for RD is also limited [5].

A limitation of this case series is that we began prospectively assessing MSK knee pathology (including diagnostic US imaging) in all patients with RD seen in our interdisciplinary movement disorder clinic after we identified a patient with RD who also had an antalgic gait and endorsed a 2-month history of episodic knee pain. For the past 4 years, we have systematically evaluated all patients with RD seen in our clinic, regardless of the phenotypic subtype, to assess the incidence of knee joint pathology (effusions, meniscal injuries, ligament pathology, tendinopathy). We have since identified an increased incidence of knee effusions in patients with the stiff knee gait subtype compared to other RD subtypes.

Future studies should evaluate the incidence and prevalence of knee effusion across all RD subtypes to determine if stiff knee gait predisposes patients to developing knee joint pathology. Detailed kinematic studies are needed to clarify the biomechanical alterations seen in stiff knee gait and the compensations arising from these alterations; and to correlate MSK injuries with these alterations and compensations. While this study focused on the knee joint specifically, future studies could examine the effects of stiff knee gait on other lower extremity joints.

## 5. Conclusions

RD is a rare form of focal, task-specific lower extremity dystonia that affects elite runners, typically decades into their careers. Athletes with stiff knee gait RD appear to be at increased risk of developing secondary/acquired knee joint pathology. This includes runners with dystonia and limited KE, KF, or PF-KE coupling. Altered gait and knee joint biomechanics may lead to MSK pathology or injuries to the ipsilateral and contralateral lower-limb joints. Failure to address MSK pathology in RD may result in sub-optimal treatment. It is paramount to underscore that pain is a known trigger for increased dystonic symptoms. Therefore, unmanaged MSK-related pain may compromise treatment outcomes in RD [13].

Timely recognition of RD is required to facilitate appropriate specialty referral to evaluate this complex and, at times, multisystem condition. Confirmation of an RD diagnosis is not limited to movement disorder neurologists. Other practitioners, including MSK physicians (physiatry, sports medicine, orthopedics, primary care), nurse practitioners, physician assistants, physiotherapists, and athletic trainers, are often the first clinicians to encounter patients with suspected RD. Ultimately, an interdisciplinary, team-based approach is crucial for the diagnosis and management of RD and its secondary impairments to ensure comprehensive evaluation and targeted treatment, while avoiding unnecessary tests or interventions. MSK-US is a promising tool for the evaluation and treatment of RD. It provides high-resolution images for both diagnostic and procedural guidance and is increasingly accessible at the bedside. This technology empowers clinicians to make accurate and cost-effective MSK diagnoses at the point of care and reduces referrals for more costly and time-consuming imaging modalities, such as MRI [29].

A standardized MSK evaluation for patients with RD can guide clinical decision-making for the managing practitioners. The initial MSK exam establishes a functional baseline for comparison and helps to determine what findings are pre-existing or new when symptoms present. MSK issues can limit function and trigger dystonia. In this case series, we demonstrated that the MSK issues associated with RD are varied and can occur in either the dystonic or non-dystonic limb. Thus, a complete bilateral assessment of all underlying MSK issues must be acquired before developing a successful interventional program.

Greater clarity in diagnosing and treating MSK issues in RD will likely come from future studies investigating the relationship between altered ground reaction forces, joint kinematics, and knee pathology in patients with RD. More importantly, future studies investigating the optimal sequence of interventions for patients with concomitant RD and MSK injuries are crucial for developing effective and personalized treatment algorithms for each patient with RD.

## Figures and Tables

**Table 1:** RD phenotypic subtypes.

**Foot/Ankle**
Toe Curling
Equinus
Inversion
**Knee**
Flexion (Hamstring Dystonia)
Extension (Quadriceps Dystonia)
Plantarflexion-Knee Extension Couple
**Hip**
Thigh Adduction
Internal Rotation

**Table 2:** Case Reviews.

**Case 1: Clinical Course**	
Initial symptoms	Right knee stiffness with progressive difficulty running.
Symptom progression	Worsening right knee stiffness and pain with running, that rapidly progressed to affect walking. Time of symptoms to diagnosis: 4 years.
Evaluation	Physical therapy (PT) evaluation: 4 years from symptom onset. Movement disorder neurologist referral: RD diagnosis.
Interventions/treatments outside NIH	Traditional PT: limited improvement. Arthrocentesis & US-guided corticosteroid injection to right popliteus tendon.
Referral to NIH	Motion analysis: right stiff knee gait, PE-KE coupling. Physical exam: right knee effusion, tenderness to palpation of posterolateral joint line, varus laxity.
Diagnostic US	Two-dimensional US: right Baker’s cyst and large lateral suprapatellar recess fluid collection (Figure 1).
Diagnostic lidocaine blocks	Right rectus femoris selective femoral nerve block: no improvement. Right tibial motor point blocks medial/lateral gastrocnemius: modest improvement on motion analysis.
Botulinum Neurotoxin (BoNT) treatments at NIH	Initial and Current: right medial and lateral gastrocnemius muscles.
Current functional status	Improved ambulation with walking, but no return to running. Cycling without impairment. Continued BoNT injections in 3–4-month intervals. Duration of NIH follow-up: 4 years.
**Case 2: Clinical Course**	
Initial symptoms	Intermittent, excessive, out-of-phase left ankle PF with treadmill running, appreciated only by patient. Time of symptoms to diagnosis: 8 years.
Symptom progression	Worsening abnormal PF and difficulty with toe clearance notable to spouse (5–7-year time-period). Progressed to foot equinus limiting running and walking. Backwards run/walk less affected. Able to cycle and swim with no symptoms.
Evaluation	PT and orthopedics referral (3–5 years post symptom onset). General neurology referral: RD diagnosis.
Interventions/treatments outside NIH	PT and AFO: no benefit. AFO worsened symptoms and caused metatarsal stress fracture. Trihexyphenidyl and carbidopa levodopa: no benefit. Initial sEMG-guided BoNT PFs: no benefit.
Referral to NIH	MRI: brain/spine: unremarkable. EDX testing was unremarkable. Spine X-Rays: scoliosis. Motion Analysis: PF-KE coupling.
Diagnostic US	Initial US: revealed left knee suprapatellar recess effusion, left Baker’s cyst. Cyst aspiration arthrocentesis: prior to BoNT injections.
Diagnostic lidocaine blocks	Left vastus lateralis/rectus femoris motor point blocks, mild benefit.
BoNT treatments at NIH	Initial pattern: quadriceps, plantar flexors—no significant improvement with addition of quadriceps. Current pattern: plantar flexors, tibialis posterior: mild-to-moderate benefit.
Current functional status	Regular BoNT injections. Ambulates (limited distance with increased speed). Unable to run. Duration of NIH follow-up: 6 years.
**Case 3: Clinical Course**	
Initial symptoms	Left leg dragging sensation, noticeable during running.
Symptom progression	Symptom onset: 18 months before referral. Walking affected: 1 year after symptom onset. Impaired balance and falls.
Evaluation	Neurologist (initial): prescribed orthotics. Orthopedic surgeon: for MSK pain. PT: minimal improvement. Movement disorder neurologist: left RD diagnosis.
Interventions/treatments outside NIH	Orthotics: left solid ankle AFO. Oral medication: baclofen, levodopa. Initial BoNT injections: tibialis anterior (TA), quadriceps femoris (no significant benefit). MRI left knee: partial medial meniscus tear, tibial plateau fracture following trial of AFO. Surgical procedures: left medial meniscus repair.
Referral to NIH	Computerized motion analysis: left PF-KE couple.
Diagnostic US	No significant findings in dystonic left lower limb; large Baker’s cyst in contralateral knee (Figure 2).
Diagnostic lidocaine blocks	Left gastrocnemius, soleus, flexor digitorum longus, and tibialis posterior (with significant improvements).
BoNT treatments at NIH	Left gastrocnemius, soleus, flexor digitorum longus, and tibialis posterior.
Current functional status	Continued significant functional improvements with walking and running. Repeat BoNT injections every 4–5 months. Duration of NIH follow-up: 11 years.
**Case 4: Clinical Course**	
Initial symptoms	Left toe clearance limited, with tripping.
Symptom progression	Two years of mild symptoms, with gradual progression to affect walking. Left toe drag and mild-to-severe abnormal plantar flexion, limiting toe clearance.
Evaluation	Neurologist: suspected left RD.
Interventions/treatments outside NIH	PT: no benefit. Foot drop AFO: no benefit; experienced mechanical fall when descending stairs.
Referral to NIH	Computerized motion analysis: left RD with PF-KE couple.
Diagnostic US	Mild left suprapatellar recess effusion and small Baker’s cyst.
BoNT treatment at NIH	Initial treatment pending. Duration of NIH follow-up: 2 years.
**Case 5: Clinical Course**	
Initial symptoms	Altered running gait on left lower extremity, resulting in left shoe clipping his right medial malleolus.
Symptom progression	Symptoms progressed during running to difficulty extending left knee in the swing phase, resulting in shortened left stride. Symptoms generalized to affect walking over several years (less severe than with running).
Evaluation	Multiple PT and orthopedics evaluations: insignificant improvement, with unremarkable MSK workup, including XR of the hip and spine, MRI spine, and 2x EDX testing.
Interventions/treatments outside NIH	PT, shoe changes, spinal manipulation.
Referral to NIH	RD, KF pattern was confirmed via observational and computerized motion analysis (7 years after symptom onset). Clipping of right ankle by left shoe was attributed to thigh adduction/KF/external rotation, based on sEMG patterns during motion analysis.
Diagnostic US	Tendinosis of right > left distal quadriceps tendon at insertion on superior pole of patella (Figure 3). Decreased stiffness of right distal quadriceps tendon using Shear Wave Elastography (SWE) (Figure 4).
Diagnostic lidocaine blocks	Not required/performed.
BoNT treatments at NIH	Initial: left semitendinosus, semimembranosus. Left adductors longus/brevis and tensor fasciae latae added. Injections in hip flexors and gastrocnemius failed to improve his gait. An injection of left tibialis posterior at an outside clinic, to address right ankle clipping, failed to improve his symptoms. Current pattern: left semitendinosus, semimembranosus, TA and adductors longus/brevis.
Current functional status	Shortened stride length on left with functionally short left lower limb in stance, due to reduced extension in swing phase and excessive flexion at initial contact through stance phase. Also noted: mild thigh adduction and excessive left ankle dorsiflexion. Gait findings attributed to dystonia in knee flexors, adductors, and TA. Duration of NIH follow-up: 18 years.
**Case 6: Clinical Course**	
Initial symptoms	Intermittent, unsustained excessive right hip and knee flexion, most notable during brisk forward ambulation on flat surfaces.
Symptom progression	Hip and knee flexion worsened with forward ambulation, but improved with slowed gait cadence. Symptoms worsened, became continuous, affecting walking at any speed, preventing him from running.
Evaluation	Primary care MD referred to general neurology, general neurologist referred to movement disorders clinic for diagnosis of RD 18 months after symptom onset. Right knee MRI showed osteoarthritis. Referred to orthopedic surgery.
Interventions/treatments outside NIH	Levodopa/Carbidopa and Trihexyphenidyl: No benefit. Initial BoNT treatments unsuccessful. Platelet-rich plasma, corticosteroid, and Hyaluronic Acid injections to right knee with significant improvement in knee pain.
Referral to NIH	Referred 2 years after symptom onset. Computerized Motion Analysis: excessive knee/hip flexion in swing and stance. Excessive right ankle dorsiflexion and foot eversion with great toe extension. EMG: continuous activity in all right hamstring muscles.
Diagnostic US	Right knee: suprapatellar lateral recess effusion, patellar cortical irregularity, deep infrapatellar effusion consistent with OA (Figure 5). Left knee: diffuse cortical irregularities consistent with OA.
BoNT treatments at NIH	Semitendinosus, semimembranosus, biceps femoris long/short heads, TA with significant symptom improvement. Initial injection interval—3 months, currently 4–5 months.
Current functional status	Forward walking symptoms improved following BoNT, symptoms increased at end of injection cycle. Elliptical with minimal symptoms. Swims and cycles with no symptoms. Unable to run. Duration of NIH follow-up: 16 years.
**Case 7: Clinical Course**	
Initial symptoms	Four-to-five-year history of progressive difficulty with right toe clearance while running. Slight dragging of right toes during running leading to several falls.
Symptom progression	Four years after symptom onset, developed stress fracture of right second metatarsal, requiring boot immobilization. Following immobilization in cast boot, her symptoms markedly increased, affecting running and walking. Symptoms expanded to include limited KF, as well as toe clearance in swing and knee hyperextension in stance. Duration of symptoms prior to diagnosis: 4.5 years.
Evaluation	Neurological examination, EDX testing, and full-spine MRI were unremarkable.
Interventions/treatments outside NIH	Extensive PT for gait problems, with no improvement. Orthopedic evaluation and cast boot for right second metatarsal stress fracture.
Referral to NIH	Self-referred to our clinic for evaluation and motion analysis. Computerized sEMG: limited right KF during swing phase, knee hyperextension during mid-stance. EMG: out-of-phase activation of vastus medialis, vastus lateralis, and rectus femoris.
Diagnostic US	Initial: right suprapatellar lateral recess effusion and bilateral patellar cortical irregularities. Repeat: large right knee suprapatellar recess effusion and small left knee suprapatellar recess effusion.
BoNT treatments at NIH	Rectus femoris, vastus lateralis, vastus medialis.
Current functional status	Improved walking, including toe clearance and KF in swing. Persistent knee hyperextension in stance. Denies knee pain, despite US evidence of knee effusions. Continues to receive significant benefit from regular BoNT injections. Duration of NIH follow-up: 3 years.
**Case 8: Clinical Course**	
Initial symptoms	Six-year history of intermittent abnormal left foot supination/inversion and toe curling when running, appreciated only by patient.
Symptom progression	Progressive worsening of toe clearance in swing phase over 6 years. Progressive shift from foot flat to outside of left foot stance. Difficulty with both inclines and declines. The symptoms remained painless and improvement in toe clearance while running backwards. Duration of symptoms prior to diagnosis: 6 years.
Evaluation	PT/runner’s clinic: ineffective. Neurology referral: established left RD diagnosis. MRI: brain and spine: unremarkable. EDX testing was unremarkable. Movement disorder evaluation in 2024 confirmed RD with forefoot supination/inversion-predominant pattern.
Interventions/treatments outside NIH	PT: no benefit. Oral medications: not trialed. EMG-guided BoNT PFs and tibialis posterior: no benefit.
Referral to NIH	Motion analysis: left forefoot supination/inversion with TA out-of-phase activity (Figure 6). Mild excessive stance-phase PF with knee hyperextension, bilaterally. KF in swing phase: within normal limits and symmetrical. Left RD diagnosis without PF-KE coupling confirmed.
Diagnostic US	Unremarkable.
Diagnostic lidocaine blocks	Not performed.
BoNT treatments at NIH	Recommended, not yet performed: TA, flexor digitorum longus, with consideration of low dose in tibialis posterior and soleus.
Current functional status	Patient self-limited running, leading to improved symptoms. Plan: continue current running schedule for 6–8 weeks following initial BoNT injections. Reassess gait pattern, and if symptoms are improved, consider slow, gradual increase in mileage and speed. Duration of NIH follow-up: 8 months.

## Data Availability

The original contributions presented in this study are included in the article/Supplementary Material. Further inquiries can be directed to the corresponding author(s).

## Supplementary Materials

The following supporting information can be downloaded at https://www.mdpi.com/article/10.3390/toxins17030121/s1: Table S1: Clinical Case Summaries.

## Abbreviations

RD	Runner’s dystonia
MSK	Musculoskeletal
MRI	Magnetic resonance imaging
EDX	Electrodiagnostic
sEMG	Surface electromyography
KF	Knee flexion
KE	Knee extension
PF	Plantarflexion
AFO	Ankle–foot orthosis
BoNT	Botulinum neurotoxin
US	Ultrasound
PRP	Platelet-rich plasma
CBT	Cognitive behavioral therapy
TA	Tibialis anterior
TBI	Traumatic brain injury
ATFL	Anterior talofibular ligament
CFL	Calcaneofibular ligament
HA	Hyaluronic Acid

## Appendix A

**Table A1:** BoNT pattern and dosing.

**Case**	**Botulinum Toxin (BoNT)**	**BoNT Injection Pattern/Most Recent Dosage**	**BoNT Response**	**Lidocaine 1% Motor Point Blocks/Response**
1	Abobotulinum Toxin A Incobotulinum Toxin A	R. Med Gastrocnemius, 100 u R. Lat Gastrocnemius, 40 u R. Soleus, 75 u R. FHL, 20 u	Fair to moderate	Prior to initial BoNT Tx: R gastrocnemius med, lat: improved gait Prior to BoNT Tx #3: R rectus femoris: no response
2	Onabotulinum Toxin A	L. Med Gastrocnemius, 80 u L. Lat Gastrocnemius, 60 u L. Soleus, 20 u L. Tib Posterior, 60 u	Fair to moderate	Prior to initial BoNT Tx: R gastrocnemius med, lat: improved gait Prior to BoNT Tx 4: R rectus femoris: no response
3	Onabotulinum Toxin A	L. Med Gastrocnemius, 35 u L. Lat Gastrocnemius, 25 u L. Soleus, 35 u L. Tib Posterior, 20 u L. FDL, 20 u	Initial response excellent Poor response x 2 in 2021 during COVID Tx delays, and guidance switched from US to EMG Excellent response from 2021 to 2024 with US guidance	Prior to BoNT Tx 10/2021 after poor response to BoNT 2021: L gastrocnemius med, lat: improved gait
4	N/A	Recommended (Not Yet Performed): L. Tibialis Anterior L. Flexor Digitorum Longus	N/A	N/A
5	Onabotulinum Toxin A	L. Adductor Longus, 70 u L. Semimembranosus, 100 u L. Semitendinosus, 100 u L. Tensor Fascia Lata, 15 u	Moderate	N/A
6	Onabotulinum Toxin A	R. Biceps Femoris, 80 u R. Semimembranosus, 80 u R. Semitendinosus, 80 u R. Tib Anterior, 20 u R. Fibularis Brevis, 20 u R. Ext Hallucis Longus, 20 u	Moderate	N/A
7	Onabotulinum Toxin A	R. Rectus Femoris, 30 u R. Vastus Intermedius, 20 u R. Vastus Lateralis, 30 u R. Vastus Medialis, 20 u	Fair to moderate	N/A

**Abbreviations**: BoNT—botulinum toxin, EMG—electromyography, Ext—extensor, FDL—flexor, R—right, L—left digitorum longus, FHL—flexor hallucis longus, Lat—lateral, Med—medial, Tib—tibialis, Tx—treatment, US—ultrasound, u—units, #—number.
