# Supplementary material for: The Relationship Between Stiff Knee Gait Runner’s Dystonia and Musculoskeletal Knee Pathology: A Case Series
Source: Toxins (Basel). 2025 Mar 3;17(3):121. doi: 10.3390/toxins17030121 (PMC11945748; doi:10.3390/toxins17030121)
Supplement: Supplementary file 1 [file toxins-17-00121-s001.zip › toxins-3457072-supplementary.pdf]

Supplementary Materials: The Relationship Between Stiff Knee Gait Runner's Dystonia and Musculoskeletal Knee Pathology: A Case Series

Jared A. Stowers, Derek S. Day, Steven Jow, Sarah Heins, Euan Forrest, Yonathan M. Assefa, Paige M. Lind, Afreen Mushtaheed, Frances T. Sheehan and Katharine E. Alter

Detailed Patient Descriptions

PF-KE Couple Predominant

Case One

The patient's initial subtle symptoms rapidly advanced to an asymmetrical gait pattern, pointed out by his running peers. Over the next 2 years, his right lower limb stiffness/pain persisted and eventually generalized to affect walking and forcing him to stop running. Four years after symptom onset, he was referred by his physical therapist to a movement disorder neurologist and was diagnosed with RD. The patient then self-referred to our clinic for evaluation. During his initial visit, right knee pain prompted diagnostic 2D ultrasound (US) imaging, uncovering a right Baker's cyst and large lateral suprapatellar recess effusion. A focused knee examination was notable for mild tenderness to palpation of the posterolateral joint line and laxity with varus stress. An MRI exam revealed a proximal popliteus tendinopathy with a partial tear. Before proceeding with botulinum neurotoxin (BoNT) injections, the patient was referred for arthrocentesis and aspiration of the baker's cyst, which did not relieve his knee discomfort. A corticosteroid popliteus injection one week later provided significant and sustained relief for his lateral knee pain, allowing PT to resume.

Based on gait evaluation pre- and post-lidocaine blocks, US-guided incobotulinum toxinA (**incoA**) injections were performed (Table C) in his right medial and lateral gastrocnemius muscles. Follow-up 3D motion analysis showed improved right KF during the swing phase and persistent right knee hyperextension in stance. He was referred to PT for gait training, and an over-the-counter leaf-spring ankle-foot orthosis (AFO) was trialed, followed by a custom hinged AFO set in 5 degrees of dorsiflexion and a plantarflexion (PF) stop. A Swedish knee cage was prescribed to reduce right knee hyper-extension with minimal improvement. His symptoms were managed with regular PT, BoNT injections, and multiple revisions to his orthoses.

Approximately one year after his initial arthrocentesis/corticosteroid injection, knee pain and swelling reoccurred, and a repeat right knee joint aspiration and corticosteroid injection was performed with similar symptom improvement. Following recovery from a right cuboid fracture, 4th metatarsal fracture, peroneus longus rupture, and partial tears of his anterior talofibular ligament (ATFL) and calcaneofibular ligament (CFL), BoNT treatment continued. The dosage was increased in the medial and lateral gastrocnemius, and the soleus was initially not injected, although it had been added in the last 2 years. This led to further improvement in his PF moment and swing phase KF but less improvement in right knee hyperextension in late stance. Repeat BoNT injections at 3-4 month intervals have led to variable improvement in his dystonia and walking gait, but he has not returned to running. Since the onset of dystonia symptoms when running, he has been able to cycle with no discernable impairments. Serial knee US examinations revealed improvement to near resolution of his suprapatellar recess effusion and persistence of a small baker's cyst.

Case Two

The patient's initial symptoms consisted of intermittent abnormal left ankle PF, only noticeable during treadmill running, then increased to over-ground running, which became perceptible to the patient's spouse. After seeing an

orthopedist, she was referred to a neurologist who diagnosed the RD. Her symptoms of abnormal left ankle PF persisted and worsened with increasing foot inversion, heightened during running. Symptoms improved with backward walking and were not present when biking or swimming. Trials of trihexyphenidyl and carbidopa/levodopa were without benefit. Initial lower limb BoNT injections targeting the gastrocnemius had limited benefit and were discontinued pending further evaluation. She also failed to improve with PT and an AFO, which caused significant pain.

The patient was referred to our clinic. Initial diagnostic US evaluation revealed a left knee suprapatellar effusion and a large Baker's cyst, with no reports of left knee pain. Before BoNT injections, the patient was referred to sports medicine for arthrocentesis. Left knee diagnostic US and MR imaging revealed a chronic lateral meniscus tear and chronic lateral patellar displacement, thought to be secondary to her remote history of recurrent lateral knee dislocations treated with a lateral retinacular release.

Based on the results of motion capture pre- and post-selective nerve blocks, our clinic's initial US-guided BoNT injections targeted the rectus femoris, vastus lateralis, and medial/lateral gastrocnemius. The patient reported minimal improvement in her gait following this injection pattern. Only the gastrocnemius and tibialis posterior were injected at the next injection cycle, with modest symptom relief. A 10-month delay in treatment occurred during the COVID-19 epidemic, during which the patient noted progressive symptom worsening.

A repeat bilateral knee diagnostic US performed 10 months after the initial exam revealed a small right suprapatellar recess fluid collection, a moderate-sized right baker's cyst, and a small parameniscal cyst on her left medial meniscus. The patient deferred repeat aspiration of the effusions. She has continued to receive regular BoNT injections, which have

moderately improved her symptoms and walking gait. She has not returned to running. The injection pattern and dosage have remained stable, targeting the left medial and lateral gastrocnemius muscles, soleus, and tibialis posterior.

Case Three

The patient's initial symptoms began during increased distance training for a marathon. Initial symptoms were described as a "dragging" sensation in her left leg during running. Her gait changes eventually became apparent to other runners, particularly as her training intensified. Symptoms progressed to affect walking, which ultimately led to imbalance and falls. Despite her symptoms, she completed a marathon. After an extensive work-up (including unremarkable brain/spine MRI and EDX testing), an external neurologist established the RD diagnosis. Following an alternative opinion from a second neurologist, an over-the-counter AFO was prescribed to decrease toe drag. Shortly after the AFO trial, she developed left knee pain. Left knee MRI showed a partially torn medial meniscus and a tibial plateau fracture. She was made non-weight-bearing by her orthopedic surgeon, and PT was prescribed with limited benefit in reducing dystonia and gait deviations. A 3rd opinion from a movement disorder neurologist attributed her symptoms to left lower limb focal dystonia consistent with RD. Trials of baclofen and levodopa were unsuccessful. Tibialis anterior (TA) and quadriceps femoris BoNT injections provided no benefit.

She was referred to our clinic for evaluation. Before motion analysis and BoNT injections, the patient underwent the recommended elective repair of her torn left meniscus. Computerized motion analysis revealed PF-KE coupling with out-of-phase activation of the left gastrocnemius, soleus, tibialis posterior, and flexor digitorum longus. Diagnostic US showed a large baker's cyst in her contralateral limb, but no significant findings were found in her dystonic lower limb. US-guided BoNT injections were performed in her left gastrocnemius, soleus, flexor digitorum longus, and tibialis posterior, leading to significant improvements in walking and running gait. She had stable symptomatic benefits with

injections at 3-4 month intervals, and her care was transitioned to a local provider. sEMG-guided injections were performed at escalating doses due to reduced clinical benefit. The patient then experienced a nine-month delay in receiving BoNT due to insurance and the COVID-19 pandemic-related issues. Because of ongoing treatment delays and her increased symptoms, she returned to our clinic. She resumed US-guided BoNT injections at the prior lower dosage, which was of excellent benefit to her symptoms. Serial bilateral knee US examinations revealed an asymptomatic persistent large right-sided baker's cyst. Given her functional improvement with BoNT injections, she opted for conservative management. Her knee effusion was not aspirated. She continues receiving regular BoNT injections at 4-5 month intervals due to sustained benefits.

Case Four

The patient's initial gait abnormalities began after a lower back injury, resulting in a several-month-long running sabbatical. Less than two years after resuming running, she started experiencing changes in her gait mechanics. She first experienced intermittent difficulty clearing her left toes, causing frequent tripping. Forward running uphill was the most challenging. She stopped running for several years, and when she resumed, her symptoms returned and worsened with a new left "foot drop," leading to multiple falls. She was referred to neurology and physiatry for evaluation. Brain MRI and EDX testing were unremarkable. Based on her history and exam, a diagnosis of suspected RD led to a referral to NIH for further assessment.

Bilateral knee US findings in our clinic included a mild left suprapatellar recess effusion and a small baker's cyst. Based on her clinical pattern and motion analysis data, a trial of BoNT targeting the PFs (left gastrocnemius, soleus) was recommended to address her PF-KE couple stiff knee gait subtype. Her workup remains pending.

KF Predominant

Case Five

This lifelong runner reported 5-7 years of progressive difficulty with running and limited left KE, resulting in a shortened stride length on the left. His symptoms began with clipping of his right medial malleolus by his left shoe, requiring him to pad his ankle. Over several months, this progressed to difficulty extending his left knee when running. He tried running on various surfaces and trialed multiple shoe changes without improvement in his symptoms. Multiple PTs and orthopedic surgeons evaluated him, and he underwent numerous imaging studies and diagnostic evaluations over subsequent years. He received alternative diagnoses, including sciatica, hip arthritis, and piriformis syndrome. Chiropractic treatment did not improve his symptoms. He was referred to a neurologist, where EDX testing was unremarkable. MRI of his spine revealed degenerative disc disease and 5mm of anterior spondylolisthesis at L5-S1. His gait symptoms were initially attributable to these findings. A 2nd neurologist opined that the MRI findings were unlikely to cause the patient's gait problems, which they believed were most likely biomechanical in etiology.

The patient's symptoms progressively worsened, eventually affecting walking. Four years after symptom onset, the patient saw a 3rd neurologist, and repeat EDX studies were reported as normal. The neurologist suspected dystonia and referred the patient to our movement disorders clinic for assessment and motion analysis. The initial neurological examination was unremarkable aside from his gait deviations, which were present after 1-2 minutes of walking and almost immediately after over-ground running. Visual observation of walking and running was evident for persistent left knee flexion and external rotation noted in the swing phase. Brain and spine imaging was unremarkable, as was repeat EDX testing.

Motion analysis revealed kinematic and sEMG abnormalities, including excessive left hip and knee flexion in the swing phase, limiting terminal KE, left knee external rotation/thigh adduction, and excessive ankle dorsiflexion in the swing phase. sEMG revealed continuous medial hamstring activation and a burst of out-of-phase activity in the left adductors. Persistent KF resulted in a short stride length, functionally short left limb, and difficulty clearing his contralateral right foot during the swing phase. Clipping of the right ankle by the left heel was attributed to slight thigh adduction with knee/hip external rotation, as there was no left foot inversion or out-of-phase activity in the tibialis posterior. Backward walking significantly improved the above kinematic and sEMG findings.

The recommended US-guided BoNT injection pattern, including the left semitendinosus and semimembranosus as well as adductors longus and brevis, resulted in modest improvement in KE and ankle clipping with sufficient benefit to allow the patient to continue running distance events, including marathons. Because of persistent hip/knee flexion injections and excessive ankle dorsiflexion, the BoNT injection pattern was expanded in different injection cycles to include the iliopsoas and gastrocnemius (secondary knee flexor action) with limited benefit. The patient sought a second opinion, where injections of the left tibialis posterior were recommended and performed with no improvement of the right ankle clipping by the left heel. The current injection pattern includes the left medial hamstrings, adductors, and tensor fascia lata muscles with moderate benefit but allowing the patient to continue running.

In recent years, in addition to his dystonia, he complained of right anterior knee pain, localized to the superior anterolateral patellar border. His knee pain persisted despite management with physical therapy and relative rest. Diagnostic US revealed a loss of the typical fibrillar pattern of the quadriceps tendon at its insertion on the superior pole of the patella, which is consistent with tendinopathy. MR imaging confirmed quadriceps tendinosis at its insertion of the patella, moderately severe on the right and mild on the left. He is considering additional treatment options as his symptoms remain persistent.

His dystonia management was complicated by a traumatic brain injury (TBI) secondary to a helmeted bike accident resulting in multiple facial fractures, an intracranial hemorrhage, and a subsequent re-bleed, which required an emergency craniotomy and drain placement. Post-injury, a new onset gait ataxia was noted in addition to his pre-morbid dystonia. His TBI-related ataxia and mild neuro-cognitive impairments have improved with recovery from the TBI in the past 3 years. The patient continues to receive regular BoNT injections to his left lower extremity. His symptoms remain stable, and he continues to run.

Case Six

A lifelong runner, the patient noted an insidious onset of abnormal movements (excessive hip and knee flexion) involving his right lower limb, most notably during running and brisk forward walking on flat outdoor surfaces. Initial symptoms were intermittent, with unsustained knee flexion and ankle dorsiflexion during the swing phase. His symptoms worsened over several months and became continuous during walking or attempts at running. He reported symptom improvement with concentration and when slow walking. He denied symptoms with cycling or swimming and only reduced symptoms on an elliptical. His past MSK medical history was notable for several minor injuries to both ankles and knees after running upwards of 50 miles weekly for several decades. He was initially seen by a primary care physician and was referred to a general neurologist who suspected a diagnosis of RD. A trial of levodopa/carbidopa and trihexyphenidyl before BoNT injections resulted in no improvement in his involuntary movements or gait deviations.

He was then referred to our movement disorders clinic for a 2nd opinion and motion analysis. His neurological examination was unremarkable other than a shortened stride length on the right with excessive knee/hip flexion, walking, or running. Brain and spine imaging and EDX testing were unremarkable. Motion analysis revealed kinematic and sEMG abnormalities, including excessive knee and hip flexion in both the swing and stance phases and continuous activity in all right hamstring muscles. Excessive right ankle dorsiflexion and foot eversion with great toe extension during the swing phase were also noted. Symptoms were task-specific, improving with backward walking but not fully correcting. A

diagnosis of RD with a stiff KF pattern was confirmed. BoNT injections in the hamstrings were recommended with improved walking symptoms, but not running. After dosage escalation, the TA was added to the injection pattern with increased benefit. A trial of BoNT in the hip flexors failed to improve his walking gait and led to difficulty descending stairs.

After several years of treatment for RD, the patient reported right lateral knee pain. His pain was persistent despite relative rest and utilization of a cane. MR imaging of his right knee was notable for moderate osteoarthritis. His discomfort improved significantly after a sequence of platelet-rich plasma injections. Diagnostic US imaging revealed a suprapatellar knee effusion and a baker's cyst in his right knee.

His dystonia management was complicated by a 10-month delay in receiving BoNT injections due to necrotizing fasciitis in his right upper limb, necessitating multiple surgeries. The interruption in regular BoNT injections for his dystonia led to a significant worsening in his symptoms, a deterioration in his gait, a new onset of dystonia symptoms when swimming and cycling, and an increase in knee pain. When he resumed his regular BoNT injections, he improved his dystonic posturing with forward and backward walking, and he no longer experiences dystonia with swimming or cycling.

KE Predominant

Case Seven

The patient reported a 5-year history of progressive difficulty clearing her right foot/toes, initially while running and then eventually while walking. Despite symptoms, she continued running up to 7 miles/day until she sustained a stress fracture of her right second metatarsal, requiring immobilization in a cast boot for six weeks. After graduating from the

boot, she noticed a marked deterioration in her gait, and she had difficulty initiating KF while running and walking. PT was ineffective. Walking became more complex and painful. She was unable to run despite multiple interventions, including acupuncture and muscle relaxants. Prior workups, including brain and cervical spine MRI and EDX testing, were unremarkable, and she was self-referred to our movement disorders clinic for further evaluation.

Her neurological examination was unremarkable, excluding her dystonia and stiff knee gait. Computerized motion analysis revealed the following kinematic changes: limited right KF during swing, knee hyperextension during mid-stance, and difficulty clearing her right toes, likely due to limited right knee and hip flexion. sEMG revealed abnormal timing (out-of-phase activation) of the vasti medialis, lateralis, and rectus femoris muscles. Abnormal timing of PF activation was also present but was less prominent than that observed in the quadriceps. Based on these findings and her history, she was diagnosed with KE stiff knee RD. A bilateral knee US revealed a right suprapatellar lateral recess effusion and bilateral patellar cortical irregularities. The patient denied knee pain. Based on her history, examination, and evaluations, she was ultimately referred for BoNT injections in the rectus femoris, vastus lateralis, and vastus medialis.

Six weeks after BoNT injections, the patient was re-evaluated and reported significantly improved symptoms and walking gait. Repeat motion revealed a persistent stiff, correct knee pattern, predominantly KE dystonia but with improved KF at terminal stance-swing initiation. Repeat bilateral knee USs showed a persistent moderately large right knee suprapatellar recess effusion and a small left knee suprapatellar recess effusion. She denied focal knee pain. Therefore, the referral for aspiration was deferred. She continues to receive BoNT in her rectus femoris, vastus lateralis, and vastus medialis. She has mild to moderate improvements in her symptoms and gait with increased KF and toe clearance in the swing phase.

Forefoot inversion pattern without PF or Stiff Knee

Case Eight

This patient's initial symptoms started in 2018 with tension in her left lower leg, difficulty clearing her left toes, and left foot inversion/supination when running. In 2023, she was referred to a neurologist and received an RD diagnosis. She received multiple BoNT injections in her left gastrocnemius and tibialis posterior with minimal benefit other than a sense of improved coordination with one injection. With running, her symptoms are less severe than in the past, which she attributes to reduced running distance and speed work. Symptoms are described as a sensation of tension in her toes and posterior calf when running. She also notes some minimal symptoms when walking.

Neurological examination in our movement disorders clinic, brain/spine imaging, and EDX testing were unremarkable. Observational over-ground gait assessment revealed decreased left foot dorsiflexion and bilateral knee hyperextension (left>right) in the stance phase with no left foot supination. A slight asymmetry in left foot eversion, compared to the right, was noted in the swing phase. No apparent involuntary movements with backward walking or toe-tapping were appreciated. Computerized motion analysis during over-ground walking before treadmill running revealed excessive knee hyperextension bilaterally while standing and symmetrical left foot inversion during the swing phase. Backward walking was symmetrical. sEMG activity revealed nearly continuous activation in the left TA in the stance and swing phase with symmetric phasic recruitment in the tibialis posterior and PFs. After 10 minutes of treadmill running, increased left foot inversion and supination were observed, and the patient reported curling of her toes and tension in her lower leg. This correlated with a marked increase in left foot inversion and out-of-phase activation in the left TA. Slightly prolonged activation of the soleus was also noted bilaterally.

Based on the history, exam findings, and motion analysis assessment, a diagnosis of focal left RD with the predominant pattern being forefoot foot supination from out-of-phase activity in the TA (sEMG) and flexor digitorum longus (patient's reports of toe-curling). The kinematic assessment also revealed mildly excessive stance phase PF and knee hyperextension, but these findings were present bilaterally and were symmetrical. The observed kinematics in this patient were inconsistent with the stiff knee pattern seen in RD with PF-KE coupling caused by PF-predominant dystonia. This case illustrates the differences between patients presenting with a PF-predominant pattern of RD and those with dystonia primarily affecting muscles that act to invert/supinate the forefoot.

Diagnostic US of both knees revealed no evidence of a suprapatellar knee joint effusion, Baker's cyst, ligament, or patellar/quadriceps tendon pathology. This case revealed prominent forefoot supination and minimal PF-KE coupling.

Based on the patient's symptoms and motion analysis results, US-guided BoNT injections were performed in the TA, tibialis posterior, soleus, and flexor digitorum longus.

Table S1. Clinical Case Summaries.

Case	Dystonia Subtype	Affected Limb	Age Sex	Years of Symptoms Before RD Diagnosis	Specialists Seen Prior to RD Dx	Other MSK Pathology	MSK Interventions	Prior Dystonia Treatments	Ultrasound Findings (Affected Limb)	Ultrasound Findings (Unaffected Limb)
1	PF_KE Couple	Right	68 Male	4	• Orthopedic Surgery	• Partial Tear of R Popliteus Tendon (MRI)<br>• R Cuboid and 4th Metatarsal Fractures<br>• Peroneus Longus Rupture<br>• Partial Tear of ATFL and CFL	• MSK PT<br>• Arthrocentesis (2x)<br>• Corticosteroid Injections (2x)<br>• AFO<br>• Swedish Knee Cage Brace	• N/A	• Suprapatellar Recess Effusion<br>• Baker's Cyst	No Significant Findings
2	PF_KE Couple	Left	63 Female	10	• Orthopedic Surgery<br>• General Neurology	• L Lateral Patellar Displacement (MRI)<br>• L Chronic Lateral	• MSK PT<br>• Arthrocentesis	• Trihexyphenidyl<br>• Carbidopa/Levodopa	• Suprapatellar Recess Effusion<br>• Baker's Cyst<br>• Parameniscal Cyst	• Suprapatellar Recess Effusion<br>• Baker's Cyst

						Meniscus Tear (MRI)			(Medial Meniscus)	
3	PF_KE Couple	Left	47 Female	5	• Orthopedic Surgery<br>• General Neurology	• L Medial Meniscus Tear (MRI)<br>• L Tibial Plateau Fracture (MRI)	• MSK PT<br>• AFO	• Baclofen<br>• Levodopa	• No Significant Findings	• Baker's Cyst
4	PF_KE Couple	Left	57 Female	2	• General Neurology	• N/A	• N/A	• N/A	• Suprapatellar Recess Effusion<br>• Baker's Cyst	• No significant Findings
5	KF Pre-dominant	Left	74 Male	5-7	• General Neurology<br>• Physical Medicine and Rehabilitation	• L Patello-femoral Pain Syndrome	• MSK PT<br>• Biofeedback<br>• Acupuncture<br>• CBT	• N/A	• No Significant Findings	• No Significant Findings
6	KF Pre-dominant	Right	71 Male	2	• Movement Disorder Neurology	• R Knee Osteoarthritis	• Corticosteroid Injection (2x)<br>• Platelet Rich Plasma (2x)	• Levodopa/Carbidopa & Trihexyphenidyl	• Suprapatellar Recess Effusion<br>• Baker's Cyst	• No Significant Findings

7	KE Pre-dominant	Right	54 Female	5	• Orthopedic Surgery	• R 2nd Metatarsal Stress Fracture		• Lorazepam	• Suprapatellar Recess Effusion<br>• Cortical Irregularities	• Suprapatellar Recess Effusion<br>• Cortical Irregularities
8	Forefoot supination/inversion	Left	44 Female	6	• General Neurology	• N/A	• PT/Running Clinic	• N/A	• No Significant Findings	• No Significant Findings

Abbreviations: AFO ankle foot orthosis, KE knee extension, KF knee flexion, MSK musculoskeletal, PF_KE plantar flexion knee extension, PT Physical therapy, PRP platelet rich plasma, CBT Cognitive Behavioral Therapy, MRI Magnetic Resonance Imaging.
